# Merkel cell carcinoma in the hand. Report of two cases

**DOI:** 10.1080/23320885.2021.2025378

**Published:** 2022-01-21

**Authors:** Felipe Mesa, Marcela Cardona, Carolina Mesa, Rodrigo Restrepo, Juan Andrés Echeverri

**Affiliations:** a Titular Professor of Plastic Surgery, CES University, Medellín, Colombia; b Plastic Surgeon IQ interquirofanos, Fundacion Colombiana de Cancerologia, Clinica Vida, Medellin, Colombia; c Dermatology, Universidad Nacional de Colombia, Bogotá, Colombia; d Dermatology, CES University. Medellin, Colombia; e Dermatopathologist, CES University, Medellin, Colombia; f Physician CES University, Medellín, Colombia

**Keywords:** Hand tumor, Merkel cell carcinoma, trabecular skin cancer

## Abstract

Merkel-cell-carcinoma of the hand is rare. The Pathological and Immunohistochemical diagnosis helps us to focus the treatment. Immunotherapy has shown beneficial effects in unresectable/advanced/metastatic stages. The quantification of antibodies against Merkel-cell-polyomavirus (MCPyV) can be a useful for prognosis and follow-up. A wide margin in surgery and the sentinel node are the first option with Radiotherapy.

## Introduction

The Merkel cell was described by Friedrich Sigmund Merkel in 1875, initially named them Tastzellen cells (touch cells), who described it as a specialized sensitive skin cell for superficial touch [1,2]. They are located in the basal layer of the epidermis, with higher density where there is no presence of hair follicles such as the soles, palms and lips [3]. Its nucleus is ovoid and its cytoplasm contains granules with neuroendocrine substances [4].

Merkel cell carcinoma (MCC), also known as trabecular carcinoma of the skin, occurs mainly in patients older than 50 years, males, Caucasians, people with immunodeficiencies, Merkel cell polyomavirus (MCPyV) infection, and exposure to ultraviolet rays [3,5]. MCPyV is a virus with oncogenic potential that interacts with p53 and induces infections in immunocompromised patients. The positive and negative MCC viruses have an immunogenic behavior and are capable of inducing responses of CD4+ and CD8+ T lymphocytes. The etiopathogenesis of virus-negative MCC would be related to a mutational load induced by ultraviolet radiation.

The most commonly affected areas are the head, neck and upper extremities [5–7], with the trunk and genitalia the least affected [8]. It has a high local recurrence and metastasis to regional and systemic lymph nodes [9].

Clinically, it has the appearance of a red-purplish, asymptomatic and rapidly growing intradermal nodule (weeks to months).

The tumor was described by Toker in 1972 as a rare and highly aggressive neuroendocrine skin cancer in the hand [1]. Then in 1978, by electron microscopy, Tang and Toker identified membrane-bound neurosecretory granules of malignant carcinoma cells [10]. Immunohistochemical studies were subsequently carried out, where positivity was found for markers such as cytokeratin 20, synaptophysin and neuronal specific enolase, thus corroborating the neuroendocrine origin and the association of the Merkel cell with the entity [1,4,5,10].

We present two cases of MCC in the second and fourth fingers of the proximal phalanx of the hand of a 67-year-old man and a 68-year-old woman.

## Results

### Case 1

**Figure 1. MCC in the first interdigital space of the left hand.**
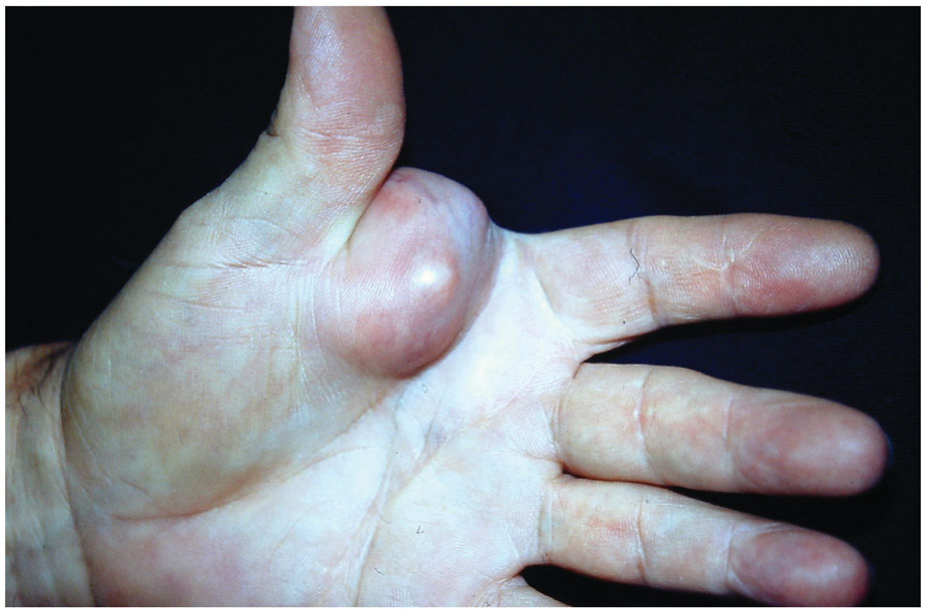


A 67-year-old man with a 2-year with a 7 × 5 cm mass in the first interdigital space of the left hand attached to the base of the index finger, which caused pain and functional disability (Figure 1).

**Figure 2.**
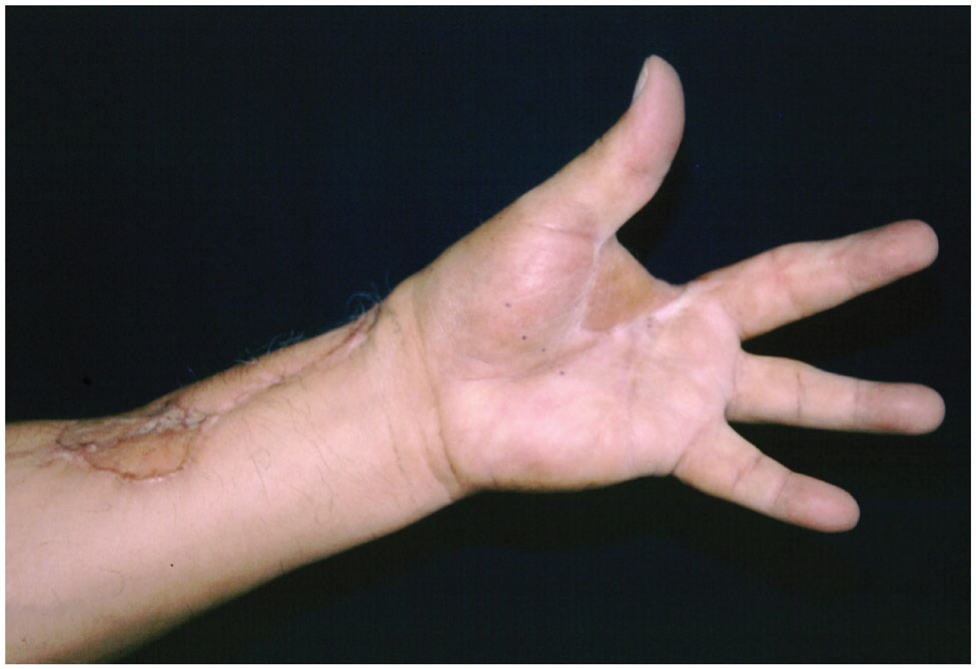
Reconstruction with a reverse radial flap.

After diagnosis by biopsy, amputation of the ray of the second finger and first interdigital space was performed, leaving proximal safety margins of 3–5 cm. Its reconstruction was performed with a reverse radial flap, to avoid retraction of the first space (Figure 2). Extension studies of the disease were performed during surgery but no metastatic lesions were found in the lymph nodes.

The pathology reported a lesion in the dermis and soft tissues of cells of mature size, vesicular ovoid nucleus, high mitotic rate, with a trabecular adenoid pattern and fibrous and hyaline stroma, with areas of necrosis. Immunohistochemistry showed positivity for cytokeratin 20 and neuronal specific enolase and negativity for chromogranin, vimentin, and epithelial membrane antigen. Synovial sarcoma was ruled out, favoring adnexal carcinoma like Merkel cell carcinoma.

He received treatment with radiotherapy three weeks after the surgery. Controls every 3–6 months for 3 years were normal. The patient died three and a half years later from cardiovascular disease without finding metastatic lesions.

### Case 2

**Figure 3.**
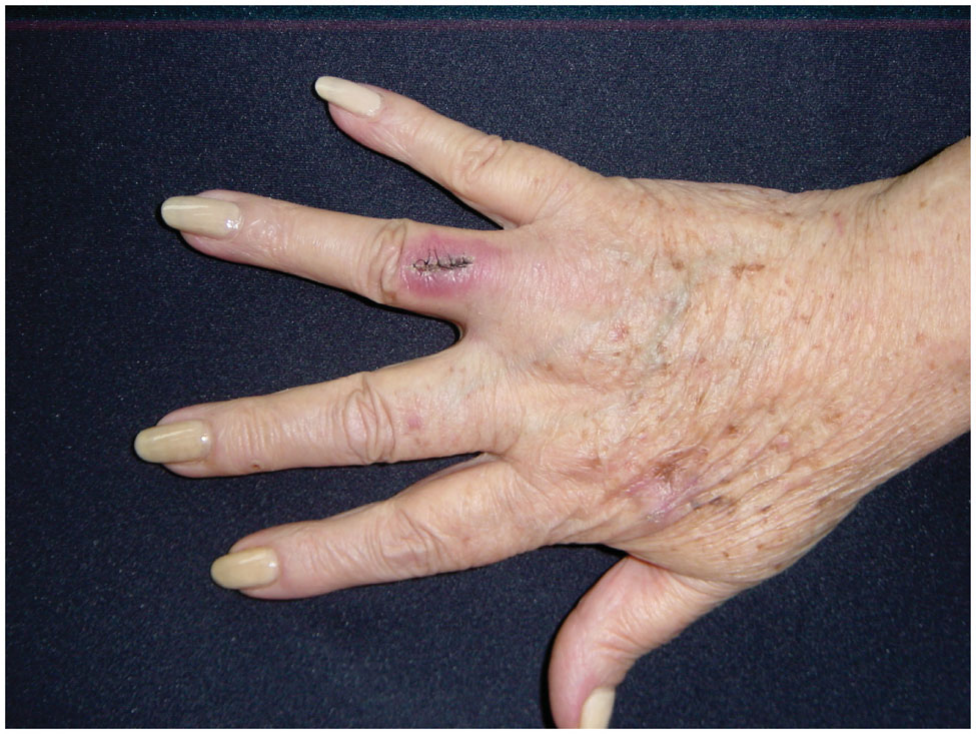
MCC on the fourth right finger.

**Figure 4.**
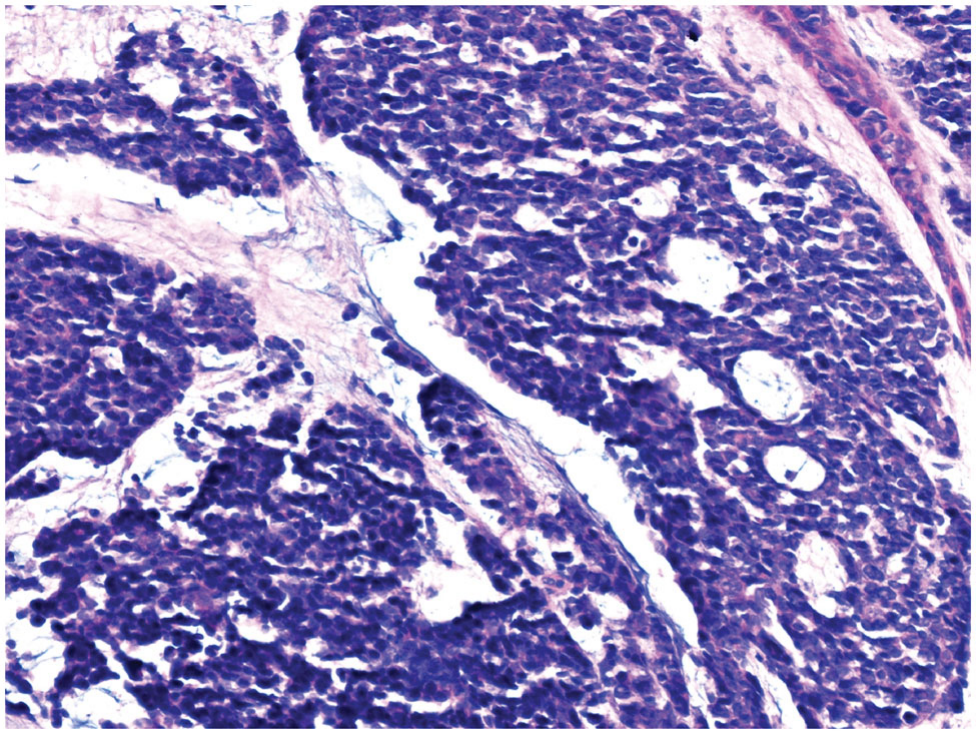
Microscopic photograph at medium magnification (HE 20×) showing basaloid cells with dense chromatin, without nucleolus. There is a slight accumulation of mucin.

**Figure 5.**
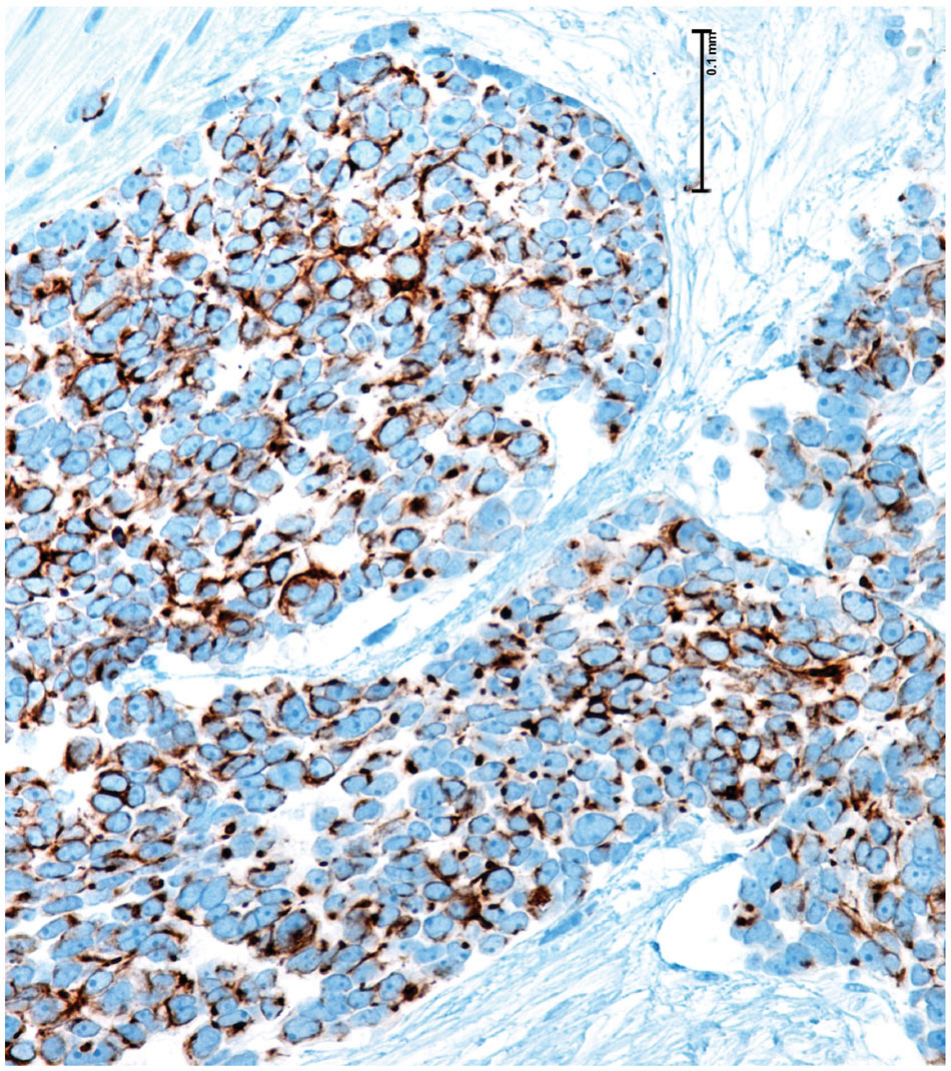
Positivity for cytokeratin 20.

**Figure 6.**
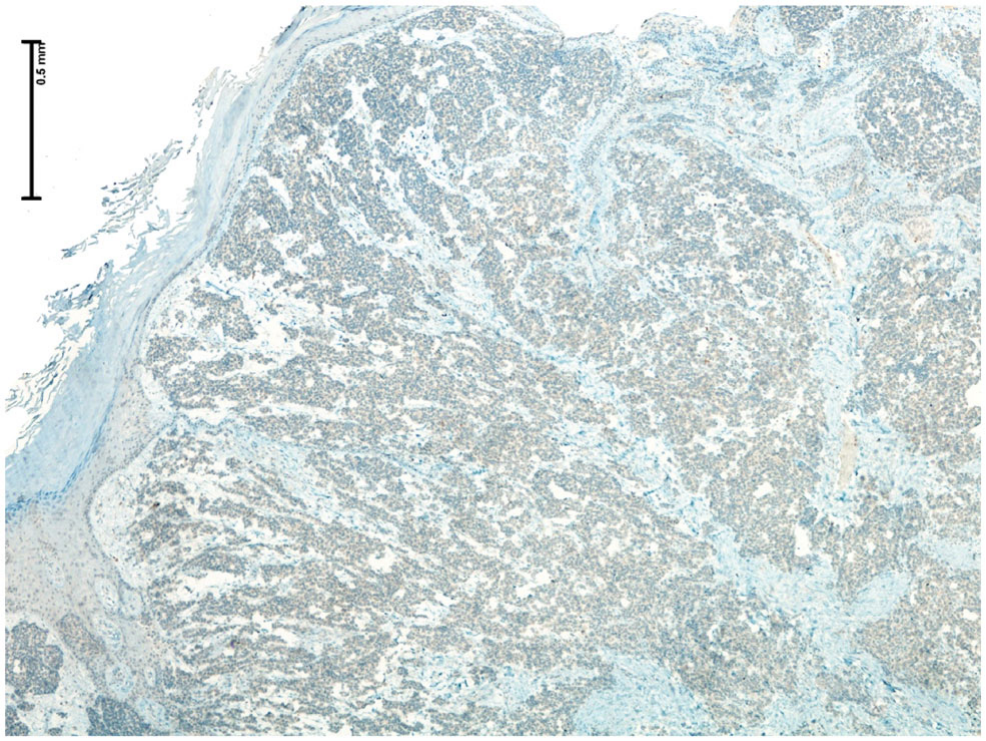
Low magnification microscopic photograph (4× IHC) showing complete negativity for TTF-1.

68-year-old female, with a mass on the dorsum of the proximal phalanx of the right fourth finger of 1 × 0.8 × 0.8 cm, slow growth, asymptomatic and purple in color (Figure 3). The biopsy reported a malignant soft tissue lesion, characterized by masses of cells with a basaloid appearance with a vesicular nucleus, without nucleolus and a pattern of fine chromatin in salt and pepper, accompanied by scant cytoplasm and with a high mitotic rate, separated by tissue trabeculae. Connective and with areas of necrosis (Figure 4), immunohistochemistry showed intensely positive cytokeratin 20 in a cytoplasmic spot staining pattern, negative cytokeratin 7, and intensely positive synaptophysin (Figures 5 and 6).

**Figure 7.**
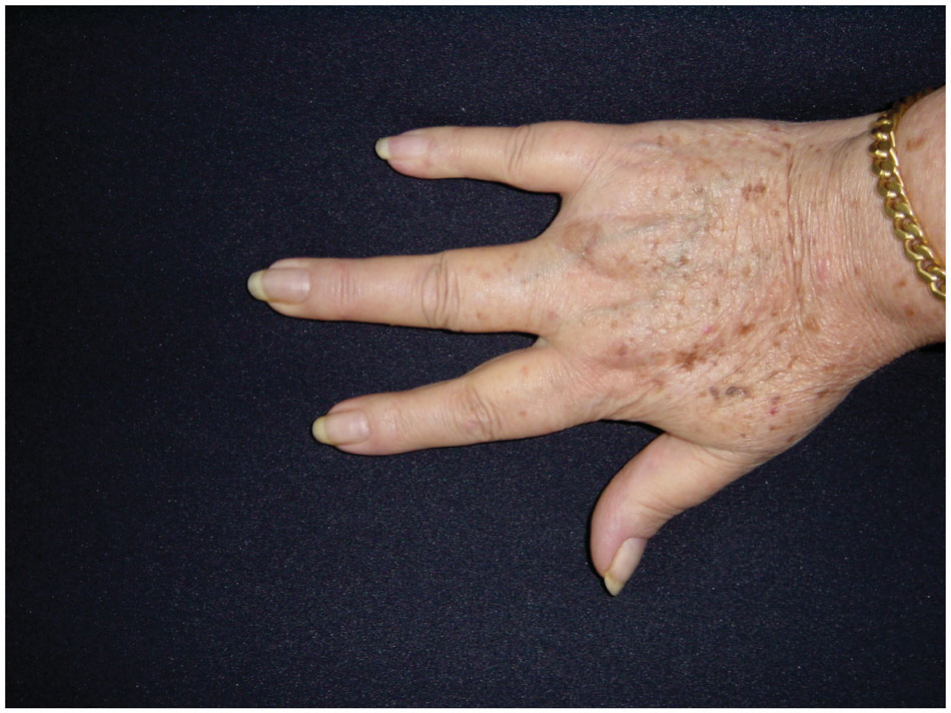
Amputation of the fourth right finger.

The fourth finger was amputated from the middle third of the metacarpus with 3-cm margins at the proximal level (Figure 7), the biopsy reported skin free of tumor infiltration in the resection margins in the area of ​​the previous biopsy, and only scar changes were reported in the previously resected area without any finding of residual tumor. Furthermore, the sentinel node study was negative. With these findings of pathology, Oncology did not consider complementary treatment at that time and radiotherapy was not performed on this occasion either. The patient was asymptomatic for 3 years and she reported that she began to notice the appearance of a rapidly growing mass in the epicondyle of the upper right limb, so she consulted for a blepharoplasty and evaluation of the mass that she did not like because It bothered her and she thought it was a lipoma so she didn’t pay attention to it and it already reached a diameter of 6 × 4 cm. The patient underwent surgery to resect and study the mass that corresponds to adenopathy, the biopsy reported a Merkel cell carcinoma, and the immunohistochemistry was confirmed by uniformly positivity for cytokeratin 20 and chromogranin intensely positive and negativity for TTF-1. Bone scintigraphy reported pathological deposits of the radiopharmaceutical in the right humerus, left shoulder, middle third of the right femur, distal third of the left femur and in L5 hemibody, suggesting metastatic lesions. She died of the disease 4 years after the initial diagnosis.

## Discussion

Merkel cell carcinoma of the hand is an aggressive and rare condition. It affects the upper limb in 20%, having a greater predilection for the head and neck (27–35%) [11,12]. Tumors on the dorsum of the hand are rare and increase in relation to exposure to UV radiation [3].

A recent study that included 492 patients with primary cutaneous MCC without apparent initial metastatic involvement, demonstrated that the performance of imaging tests (CT, MRI or PET/CT) in the initial staging of the patient identified distant metastases in 4, 3% of the cases. In addition, 13.2% required reclassification to more advanced stages. These results justify performing imaging tests even in patients without clinical evidence of lymph node involvement or metastatic disease or with negative selective sentinel node biopsy (SLNB) [13].

Correct staging of MCC is essential to determine prognosis and therapeutic behavior. It is reported in the literature that at the time of diagnosis the regional lymphatic involvement is from 35.4% to 65%, distant metastasis from 13.5% to 40% and after surgical treatment there is local recurrence between 25% and 35% [5,9,12,14]. With a mortality between 33%–46% and a 5-year survival that varies according to whether 64% present local disease, 39% lymph node involvement, or 18% distant metastasis [5].

Soltani et al., found that patients with MCC in the hand and upper limb had a lower frequency of lymph node involvement at diagnosis and a longer survival, possibly due to early diagnosis. The prognosis is inversely proportional to the size of the lesion [9].

Regarding treatment, the surgical margins of tumor resection accompanied by radiotherapy have been studied by some authors and no significant differences were found in the survival of the disease between margins of 1–2 cm [15].

Sentinel lymph node study is important in these patients, since in up to 30% of cases regional lymphatic metastasis may be present in early stages [16].

Other treatments that are being studied with good results are PD-1/PD-L1 inhibitors and they have revolutionized the treatment of metastatic MCC. In 2017, Avelumab was the first drug to be approved for several regulatory agencies for the treatment of metastatic MCC. More recently, Pembrolizumab has also been approved by the U.S. Food and Drug Administration in this setting. Immunotherapy has therefore become the new standard of care in advanced MCC, displacing classical chemotherapy [7].

In recent years, the role of MCPyV has gained special importance in the pathogenesis of this tumor. The quantification of MCPyV antibodies can be a useful tool as a prognostic indicator and in follow-up, allowing the early detection of recurrences. Our two patients were not performed because they were from very basic health entities where this study was not allowed as a diagnostic complement due to costs.

Our first case had a late diagnosis (2 years) and was disease free for 3.5 years before dying. While the second had several months of evolution with a tumor size of 1 cm and died of the disease 4 years after the initial diagnosis. This indicates how aggressive the pathology is and how much the behavior of the tumor is unknown.

Diagnosis by pathology is made with the presence of basaloid cells, vesicular nucleus, high mitotic cup, separated by trabeculae of connective tissue and areas of necrosis. Immunohistochemistry showed positivity for cytokeratin 20, neuron specific enolase and negativity for chromogranin, vimentin, and epithelial membrane antigen in the first case and for cytokeratin 20 in a cytoplasmic spot staining pattern, intensely positive synaptophysin and negative cytokeratin 7 in the second case, all specific markers of the disease [4].

With the presentation of these two cases of MCC in the second and fourth fingers of the proximal phalanx we find several data to discuss and difficult to conclude. In theory, the size of the tumor greater than 2 cm, male sex, age greater than 70 years, the histological patterns of small cells, lymphovascular invasion, can be a predictor of poor prognosis [6], but in this first patient the behavior and evolution of the disease was very mild, compared with the second case where the diameter of the tumor was only 1 cm with equally wide resection margins and no initial lymph node involvement with negative extension study examinations for the disease but perhaps without initial radiotherapy and with poor follow-up of the disease due to the patient’s non-participation in the medical consultation until the appearance of the mass in the elbow region, when the metastasis was found and the rapid evolution that led to death. With the findings found in the literature, it is also important to take into account the study of the quantification of MCPyV antibodies as a prognostic value.

These clinical cases seek to sensitize those who diagnose and treat them for the aggressiveness of the disease and the importance of defining the early oncological treatment to follow. First a timely diagnosis with biopsy, then a thorough study of imaging tests to define the extent of the disease, the study of MCPyV antibodies as a prognostic value. Subsequently, tumor resection with surgical safety margins, sentinel lymph node study and later the complementary treatment with radiotherapy and immunotherapy and chemotherapy according to Oncology. also insist on close monitoring of the patient to avoid residual and/or metastasis.
